# A novel Veress needle mechanism that reduces overshooting after puncturing the abdominal wall

**DOI:** 10.1007/s00464-021-08603-x

**Published:** 2021-06-22

**Authors:** Roelf R. Postema, David Cefai, Bart van Straten, Rein Miedema, Latifa Lesmana Hardjo, Jenny Dankelman, Felix Nickel, Tim Horeman-Franse

**Affiliations:** 1 Department of BioMechanical Engineering, Faculty of Biomedical Engineering, University of Technology Delft, Mekelweg 2, 2628 CD Delft, The Netherlands; 2 Department of Surgery, University Medical Centers Amsterdam, Location VUMC, De Boelelaan 1117, 1081 HV Amsterdam, The Netherlands; 3 Engineering Department, ProVinci Medtech, 2631 CM Nootdorp, The Netherlands; 4 Department of General, Visceral, and Transplantation Surgery, Heidelberg University Hospital, Im Neuenheimer Feld 420, 69120 Heidelberg, Germany

**Keywords:** Laparoscopy, Entry technique, Veress needle, Safety Mechanism

## Abstract

**Background:**

Complications that occur in laparoscopic surgery are often associated with the initial entry into the peritoneal cavity. The literature reported incidences of Veress needle (VN) injuries of e.g. 0.31% and 0.23%. In a 2010 national survey of laparoscopic entry techniques in the Canadian General Surgical practice, 57.3% of respondents had either experienced or witnessed a serious laparoscopic entry complication like bowel perforation and vascular injury. As those complications are potentially life threatening and should be avoided at all costs, improving safety of this initial action is paramount.

**Methods:**

Based on a bare minimum design approach with focus on function expansion of existing components, a new Safety mechanism was developed for the VN that decreases the risks of VN overshooting. The mechanism works by preventing the puncturing acceleration of the tip of the VN by decoupling the surgeon’s hand from the VN immediately after entering the abdomen.

**Results:**

Based on a set of requirements, a first prototype of the VN+ with force decoupling safety mechanism is presented and evaluated on an ex vivo porcine abdominal wall tissue model in a custom setup. The experiments conducted by two novices and one experienced surgeon indicated a significant difference between the attempts with a standard, conventional working VN (41.4 mm [37.5–45 mm]) and VN+ with decoupling mechanism (20.8 mm [17.5–22.5 mm]) of *p* < 0.001.

**Conclusion:**

A new decoupling safety mechanism was integrated successfully in a standard VN resulting in a VN+ . The results from the pilot study indicate that this new VN+ reduces overshooting with a minimum of 50% in a standardised ex vivo setting on fresh porcine abdominal wall specimens.

**Supplementary Information:**

The online version contains supplementary material available at 10.1007/s00464-021-08603-x.

Laparoscopic surgery is a widely performed technique that has replaced open surgery as the gold standard in general surgery, gynaecology, and urology [1]. In general, three types of methods are used to gain initial access to the peritoneal cavity to establish pneumoperitoneum. The first, the so-called open technique as described by Hasson in 1971, requires a direct incision through the abdominal wall prior to the trocar insertion [2]. The second, the technique using a so-called optical trocar relies on the view through the tip of a bladeless trocar whereby the different layers of the abdominal wall can be seen during introduction of this trocar, thereby visually identifying entry into the abdomen [3]. The third technique, the closed technique, uses the Veress needle (VN) to gain abdominal distention prior to the first trocar insertion [4]. Contrary to the open and optical trocar technique, the closed technique with the VN involves a blind insertion directly into the peritoneal cavity [1, 5, 6]. Combinations of the three main initial access types also exist, e.g. the VN combined with the optical trocar technique.

Complications that occur in laparoscopic surgery are often associated with the initial entry into the peritoneal cavity [5]. The literature reports incidences of VN injuries of e.g. 0.31% [1] and 0.23% [7]. An overview can be found in the Cochrane database [5]. In a 2010 national survey of laparoscopic entry techniques in the Canadian General Surgical practice, 57.3% of respondents had either experienced or witnessed a serious laparoscopic entry complication [8]. Complications like bowel perforation and vascular injury, can potentially be life threatening and should be avoided at all costs. Hence, improving safety of this initial action is paramount.

Up till now there has been no consensus on the safest method of the two techniques for initial entry into the peritoneal cavity [5]. In a study by Molloy et al. the VN was used in 81% of gynaecological laparoscopic operations and in 48% of general surgery procedures [6]. The use of the VN is probably popular because of its simplicity and effectiveness. It involves making a small incision in or near the umbilicus or in the left upper quadrant of the abdomen and then, in a blind fashion, putting the needle through the subcutaneous tissue, abdominal wall and the parietal peritoneum into the abdominal cavity. The VN technique is based on the ability of its blunt inner stylet to spring forward (spring loaded) and to cover the sharp bevelled tip of the outer cannula when resistance diminishes after all tissue layers are passed (Fig. 1). However, when the blunt tip approaches the tissue with too much speed and force, tissue damage still can occur. Hence, the surgeon cannot totally rely on this mechanism and therefore needs to develop a sense of the appropriate angle of insertion and the appropriate force to successfully puncture through the abdominal wall without overshooting into the underlying organs. The risk of damaging the underlying tissue with the tip of the cannula becomes high, when the reaction force that is generated by the abdominal wall drops to nearly zero (inside the abdominal cavity) in an instant. This immediate loss of resistance on the tip of the VN after puncturing causes acceleration of the needle towards the underlying tissues due to the slow reaction of the human control system [9, 10], absence of stiff lower arm/hand support and relatively large mass of the arm [11, 12]. Therefore, skilled and safe use of the VN requires a long learning curve to achieve the best possible instrument handling to prevent overshoot. Moreover, every patient’s abdomen presents unique operating conditions, of which the specifics are unknown to the surgeon prior to the operation. These include the presence of adhesions, positions of the underlying tissues and viscera and the thickness of the abdominal wall.

**Fig. 1:**
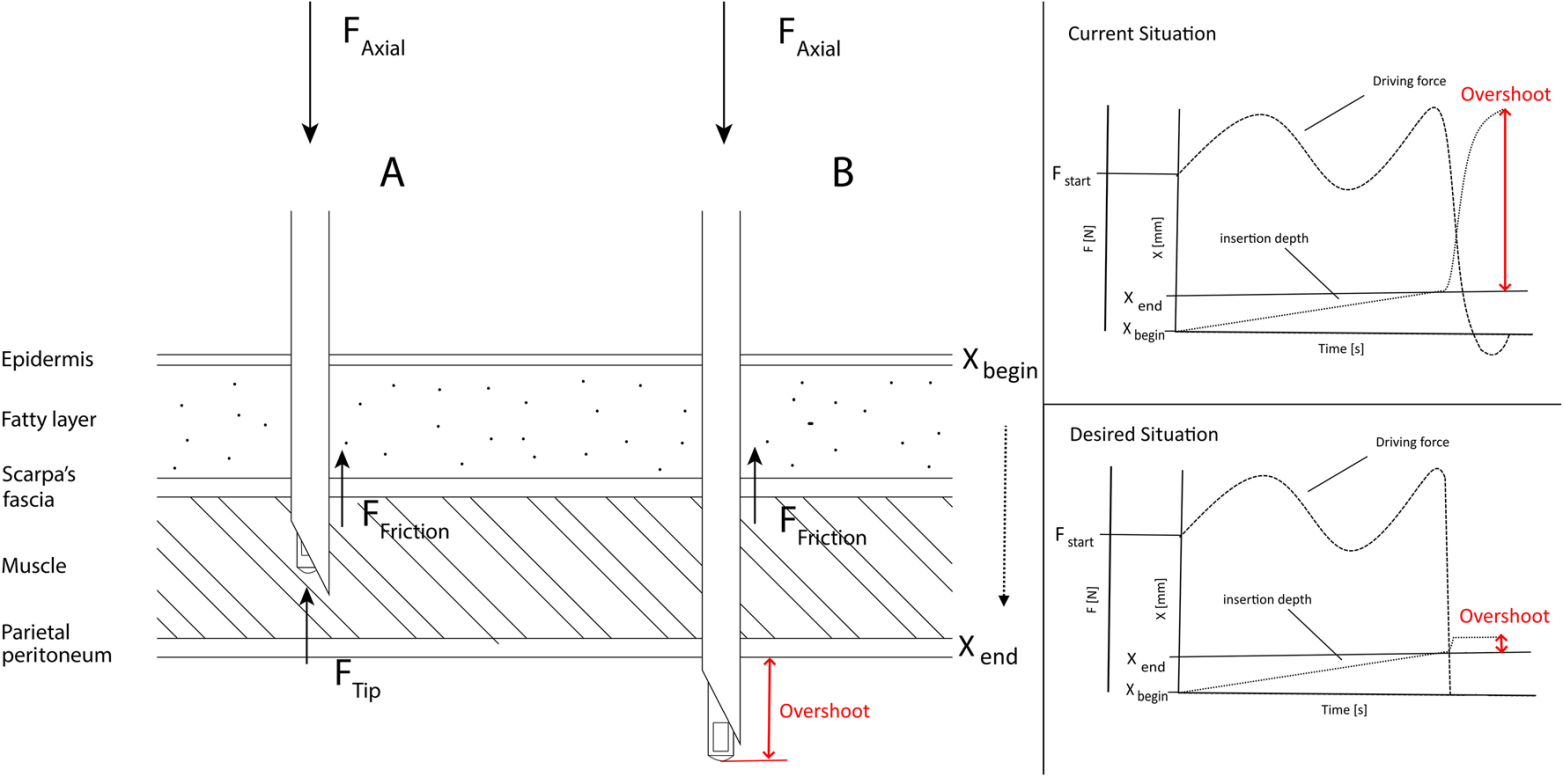
Schematic figure of the Verres needle (VN) technique and the layers of the abdominal wall. Left: In **A** the sharp bevel tipped cannula and blunt stylet are penetrating the abdominal wall. In **B** the parietal peritoneum has been punctured and the tip and stylet are in the abdominal cavity. Right upper graph: Typical driving force moving the needle further into the cavity after penetrating the last layer. Right lower graph: Desired driving force (dashed lines), reduced immediately to zero after penetrating the last layer not moving the needle further into the cavity, this limits the insertion depth (dotted lines). The amount of overshoot is indicated in red (Color figure online)

## State-of-the-art

To solve the problem of puncturing underlying tissue during VN insertion, some straightforward modifications were suggested that limit overshooting by allowing the surgeon to set the maximal insertion depth [13]. Unfortunately, the tissue layer thickness is not always known and layers are flexible making adjustment of the insertion depth difficult. Another approach was to allow overshooting but to fixate the Veress mechanism as soon as it shoots in position [14]. However, even a blunt stylet with locked outer cannula can damage internal structures. Some research groups focused on expanding the blunt area of the tip directly after insertion to reduce the stress when the tip hits underlying organs or structures [1, 15]. Other work demonstrates the capability to acquire information about intracorporeal tissue tool interactions of the VN tip, utilizing acoustic emissions or optic information recorded at the extracorporeal end of the needle [16, 17]. Following a different approach, another group created a vacuum cup around the VN. When sucking the air out of the cup, the abdominal wall is lifted away from the critical structures when the VN penetrates the tissue [18]. Although good results are presented, the surgeon does lose manipulation flexibility as the configuration of the needle is fixed. Instead of changing the mechanism’s functionality, some researchers suggest that providing additional information about pressure or vacuum on a visual scale helps reduce fatal organ damage [19–21]. Unfortunately, the effect of those indicators remains unknown. Finally, overshooting can be prevented by introducing a faster control system. This can be done by linking a robot arm with haptic sensation to the VN’s body that generates the driving force [22]. Although some of those results show interesting results, the complex nature of these systems and the impact on workflow does jeopardize broader acceptance.

An alternative way to decrease the risks of overshooting of the VN is to reduce the acceleration of the tip after puncturing as quickly as possible. For this, a new mechanism needs to be developed that immediately removes the driving force (Fig. 1, right lower graph) as soon as the tip enters the cavity.

Based on a set of requirements, a first prototype of the VN+ with force decoupling safety mechanism is presented and evaluated on an ex vivo abdominal wall tissue. The experiments were conducted by two novices and one experienced surgeon.

## Methods

### Requirements

After a thorough analysis of the problem, the following requirements were set for a new safer design of the VN:

1. Decrease acceleration after the abdominal wall has been punctured.
2. Keep the original VN functions (cannula with sharp tip, blunt inner stylet and spring loaded).
3. The haptic sensation and change in workflow during insertion should not add any risk to the procedure.
4. Device must be suitable to be sterilized in the Central Sterilization Department (CSD).
5. Compatible with multiple VN designs.

#### Decrease acceleration

A force and motion analysis has been performed to understand the mechanics behind the VN needle puncture. The needle insertion procedure contains the following phases that are taken from Van Gerwen [23]; (a) No interaction; (b) Boundary displacement; (c) Tip insertion; (d) Tip, cannula and stylet insertion. The important forces are the axial forces acting on the stylet/cannula tip, and the friction forces acting on the side of the cannula. When the VN is passing through the abdominal wall, the axial force can be described as: *F*_axial_ = *F*_tip_ + *F*_friction_ (Fig. 1A). Once the last fascia and muscle layers have been punctured, the force on the stylet/cannula tip (*F*_tip_) reduces instantly to zero. At this moment *F*_friction_ is much lower than *F*_axial_ and the needle accelerates towards the underlying tissues (Fig. 1B). Because the stiff fascia requires a high force to puncture [24] and the composition of the layers as well as a number of fascia layers differ per region [25], it becomes more difficult to predict the VN’s behaviour and to prevent overshoot.

#### Keep original VN functions

The original spring action safety feature of the original VN were kept because of the simplicity of the design and surgeons already rely on this feature during use. After insertion, the VN is used to establish a pneumoperitoneum and therefore it should allow for CO_2_ insufflation to the peritoneum.

#### The haptic sensation and change in workflow during insertion should not add any risk to the procedure

The sequence in which the needle is activated, by applying pressure on the needle tip followed by movement of the inner stylet according to a known force displacement characteristic, is often programmed deep in the surgeon's haptic system. All changes that influence the workflow or haptic sensation during insertion through the different abdominal layers should be evaluated. If these properties are changed, the face and construct validity of the needle should be proven again.

#### Suitable for sterilization in the CSD

The device should be able to be cleaned and processed in the CSD within a reasonable timeframe [26].

#### Compatible with multiple VN designs

The mechanism should work with all VN needle diameters.

### Design approach

Winter et al. found that, to gain broad acceptance of a technical innovation in the medical field, a dedicated design approach is often required [27]. This can be accomplished by developing intuitive and maintenance-friendly instrumentation that can be used by surgeon’s worldwide working in both developed and poorly resourced hospitals with basic CSSDs. We have, therefore, used a “bare-minimum design” methodology, with a strong focus on component interaction analysis and adding functions to standard components [26–28], in combination with a stepwise development and evaluation plan that involves all key users coming into contact with the VN+ to create the new safety mechanism.

### Pilot study test setup

**Fig. 2:**
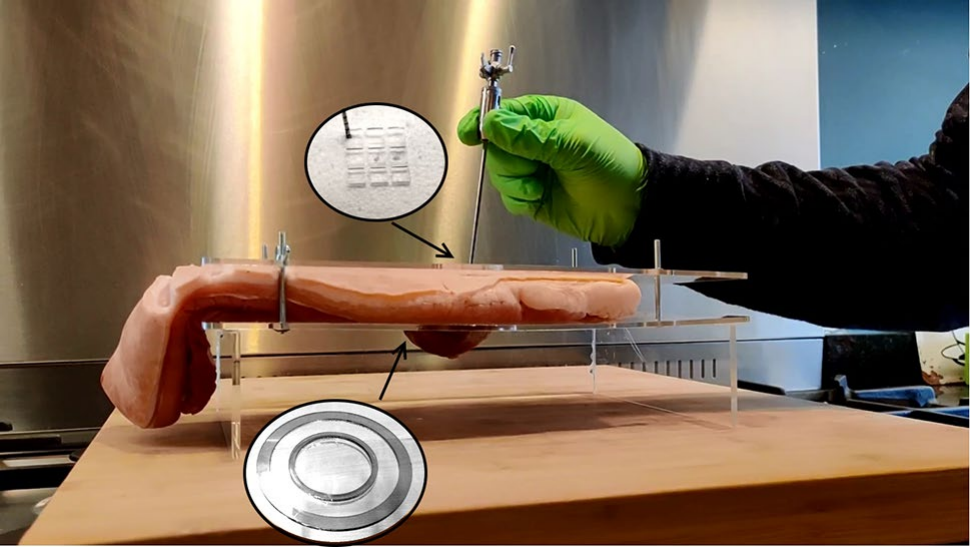
Pilot study setup consisting of a support plate with a round hole and top plate with a raster of nine square holes. The nine holes prevent that a single location on the incision is used multiple times. The round hole allows tissue to deform and the needle tip to pass. Around the round opening rims were glued to increase the grip on the tissue. Four wire taps with butterfly nuts were used to tighten the tissue between the two plates

The goal of the experiment is to compare the modified VN+ with the conventional VN when used by inexperienced users in a simulated clinical setting. The ability to puncture through the abdominal layers of a porcine wall without potentially damaging organs below the abdominal wall is determined by measuring how much of the needle penetrated the abdominal wall. For objective assessment a digital camera was used to record each measurement and to determine the lowest point of the needle cannula after the trial. A laser engraver was used to add markings on the metal of the VN cannula to determine the absolute insertion distance during use. Figure 2 shows the test setup used to fixate the specimen during the experiment. Therefore three intact abdominal walls of 200 by 150 mm were collected at the abattoir, for this no IRB approval was required. The specimens were directly frozen at -20 degrees for a maximum of three days prior to testing. Two hours before the experiments started a heating element was used to heat the samples to 37 degrees. The test setup consisted of a table with two independent Plexiglas plates of 300 by 200 mm. An opening of 60 mm was cut in the bottom plate that allowed tissue to be pressed downwards whilst following the movement of the needle tip during insertion. Two additional rims with inner diameters of 80 and 100 mm were glued on the bottom plate around the hole for extra grip on the wall after fixation. The top plate had a raster of nine square openings each, measuring 10 mm square with a 2 mm distance between each opening. The raster prevented the participant from using the same hole twice during the experiment and kept the data reliable. The sample was installed by the observer after making the first incisions in the skin. After placement of the sample on the bottom plate, the top plate was placed and loosely tightened by 4 flinders nuts. A wooden block was placed under the tissue to stabilise the setup and to prevent contact between the tip and metal table.

### Measurement protocol

To validate the setup and needle, experiments were executed by two novices and one experienced surgeon in VN placement. First, the participant got oral instructions. The experimenter explained to the participant that there are 2 modified versions of the VN, to reduce demand characteristics and bias. The participant is shown how to correctly hold the VNs and is given the task to puncture through the tissue layers and to reduce the risk of overshooting as much as possible without touching the construction of the setup. The participant was asked to put the tip of the VN on the top of the top layer in the incision and to start the procedure. It is explained not to remove pressure from the needle until the needle passed all layers and reached the “cavity”. Before the measurements started, all participants could ask questions and were allowed to investigate and test the VNs so that the principles were fully understood. Insertion of the needle was replicated 10 times with the spring-loaded safety mechanism activated (VN+) and 10 times without (VN). The participants were asked to switch the approach between each needle insertion. When all openings were used, a new tissue area was selected and the tissue relocated accordingly. Each participant used a new tissue area. In a second session, an experienced surgeon who had performed over 500 VN procedures was asked to use the needle three times with the safety mechanism activated and three times without. The surgeon was asked to position and hold the tissue as realistic as possible before inserting the needle.

### Data comparison and statistics

Video footage was used by the observer to determine the insertion distance and a screenshot was made. From this data, the number of visual markings was counted and the true insertion depth was calculated in Excel based on the known distance between each marking. After the participants completed the tests, any differences in maximum insertion depth between the “conventional” (VN) and “modified” (VN+) groups were determined with a Mann–Whitney test (SPSS v16, SPSS, Inc., Chicago, IL) as the data were not normally distributed. A *p* value < 0.05 was considered a significant difference.

## Results

### Prototype VN+ design

**Fig. 3:**
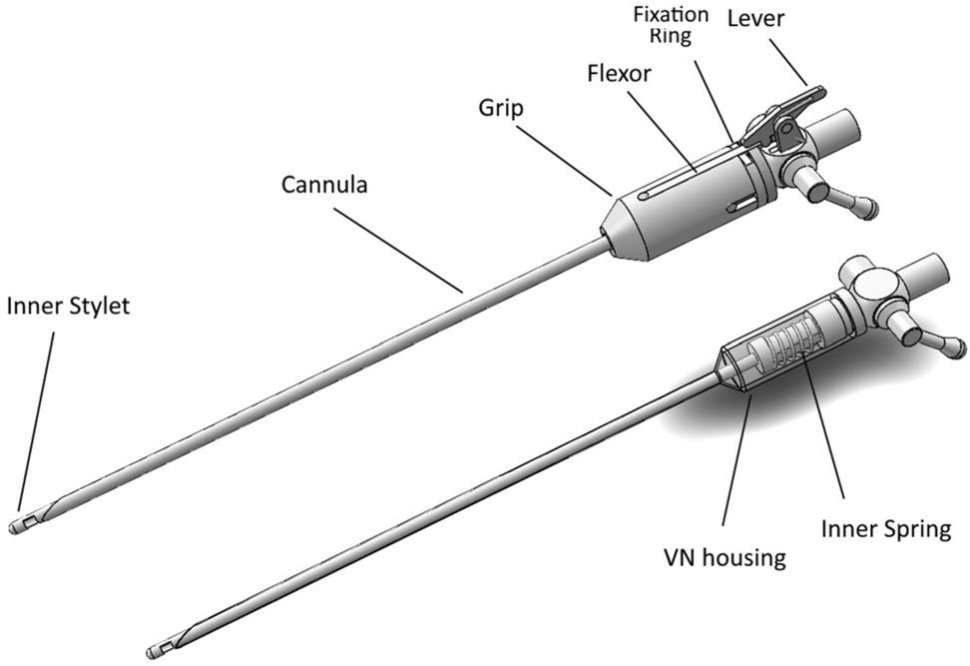
Top, VN + design with additional Lever mechanism and Grip with Flexor that slides over the modified VN housing. At the end of the Flexor a hook is welded that locks the Grip behind the fixation ring of the modified VN housing. Below, a standard VN design with transparent VN housing showing the Inner spring that applies force on the Inner stylet when it moves relatively to the Cannula

Figure 3 shows a drawing of the VN+ with new safety decoupling mechanism and a conventional VN. The VN+ (Fig. 3-Top) is based on this conventional VN (Fig. 3-Below) and has an additional lever mechanism, and grip component with flexor and some alignment pins.

**Fig. 4:**
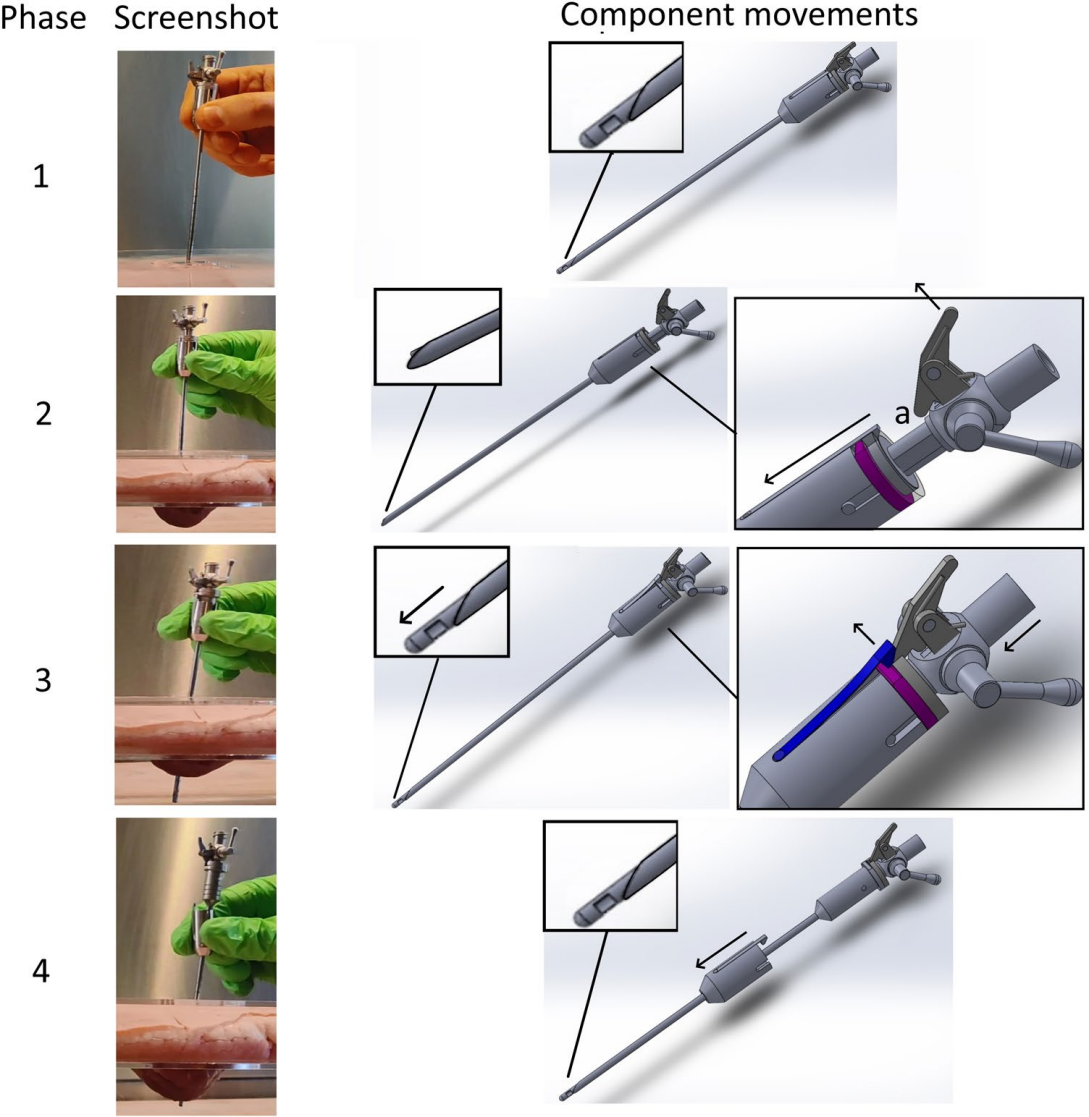
Basic functioning of the VN + design. Phase 1, the rest situation where the system is ready for use. Phase 2, during insertion the sharp cannula is exposed and the decoupling mechanism is armed automatically. Phase 3, as soon as the cannula tip penetrates the last tissue layer the stylet shoots out and decouples the grip component when the lever finger lifts the hook (dark blue) over the fixation ring (magenta). Phase 4, the force exerted by the hand on the needle is instantly decoupled resulting in acceleration of the grip component opposite to the housing (Color figure online)

Figure 4 indicates how the VN+ mechanism functions during use. In the first phase, no external forces are present on any of the components and the spring-loaded lever finger is resting on top of the hook that connects the grip component to the base of the needle. In this phase it is not possible to slide the grip component axially away from the VN+ ’s body. When the VN+ is inserted into tissue the inner stylet of the VN+ slides upwards due to the axial force on the tip in the second phase. Now the spring-loaded lever finger (Phase 2, a) moves downwards relatively to the stylet in a new resting position. In the third phase, when the parietal peritoneum has been punctured the axial reaction force on the stylet instantly drops to zero. This causes the inner spring inside the VN+ housing to deform back to its original state pushing the inner stylet back to its starting position. During this movement the finger of the spring loaded lever moves under the hook of the leaf spring (dark blue) disconnecting the grip from the VN+ . When the grip is disconnected from the rest of the needle in the fourth phase, the surgeon’s hand and grip accelerate away from the VN+ housing. Hence, the principle of the added safety mechanism is that the potential energy of the surgeon’s arm, when applying a force on the VN+ , the grip is instantly decoupled from the rest of the VN+ when the cannula tip penetrated the last tissue layer of the abdominal wall. Instead of driving the cannula further into the cavity, this potential energy is now safely removed from the needle as only the grip accelerate towards the abdomen whilst the rest of the VN+ remains in position.

**Fig. 5:**
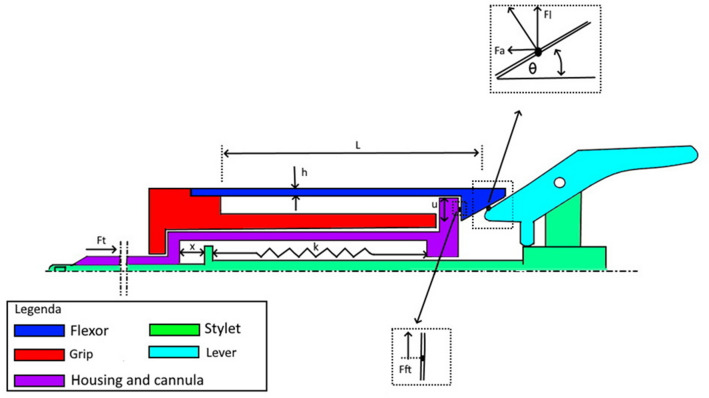
Schematic drawing of the VN + safety system. The flexor of the grip (dark blue) is lifted upwards by the lever (light blue) when the inner stylet (green) shoots to the left when all tissue layers are passed. When the flexor is lifted, the grip component (red) is decoupled from the VN + and accelerating away from the housing (Color figure online)

The working principle of the VN+ decoupling mechanism is based on differences between the cannula tip force, tissue friction force and spring force acting on the stylet. To assure fast decoupling and a strong reliable system, the flexor (Fig. 4 and 5 dark blue) should not be too thick or thin. Therefore the maximum flexor thickness was determined with Formula 1. Supplemental file 1 shows that the maximum flexor thickness can be 0.4 mm when using the standard VN spring, the flexor length and width 20 and 2 mm, respectively, the hook angle and height 30 degrees and 2 mm, respectively, and stainless steel as flexor material.

1 $$\begin{gathered} h = \sqrt[3]{{\frac{{12 \times \left( {{\text{Fl}} - {\text{Ff}}t} \right) \times L^{3} }}{{3Eub}}}} \hfill \\ {\text{Fl}} = Fa/\tan \theta \hfill \\ Fa = ~k \times x \hfill \\ {\text{Fft}} = ~\mu \times {\text{Ft}} \hfill \\ \end{gathered}$$

*h* is the thickness flexor, Fa is axial force on flexor tip, Fl is lifting force hook, Ft is friction force tissue around needle, Fft is friction force between hook and ring, *θ* is hook angle flexor tip in degrees, *L* is length of the flexor, *E* is the elasticity modulus Stainless steel, *u* is elevation hook for disconnection, *b* is width flexor, *k* is spring rate VN housing spring, *x* is spring indentation and *µ* is the friction coefficient.

Figure 5 shows a cross sectional area of the functional components of the VN+ . All relevant forces, dimensions and parameters of Formula 1 are indicated in the figure.

**Fig. 6:**
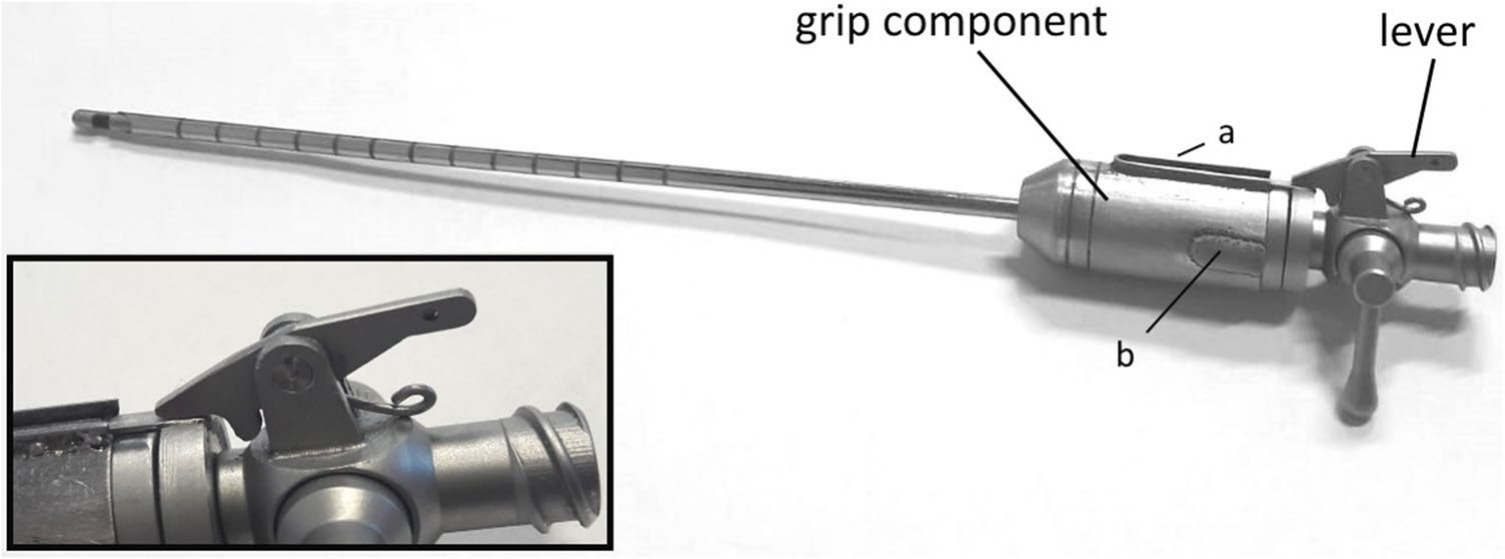
First prototype of the VN + with decoupling mechanism. Additionally, extra protection rims (a) and a cap (b) were placed on the grip component to prevent that the user interferes with the flexor element or guidance pin

The VN+ prototype in Fig. 6 was manufactured out of a standard conventional working VN needle for laparoscopic surgery made from stainless steel (SS316, TROKAMED GmbH, Kleine Breite 17, 78187 Geisingen, Germany). Modifications were done at Van Straten Medical (Rijnzathe 2, 3454PV, De Meern-Utrecht, the Netherlands). To prevent the surgeon’s fingers from interacting with the flexor or alignment pin, protective rims and covers were welded on the grip component. All extra components were made from stainless steel (SS316L) and fully accessible for rinsing. The grip component can be detached from the rest of the VN+ . The stainless steel flexor with locking hook is laser welded to the grip component.

The first prototype of the VN+ with decoupling mechanism can be found in Fig. 6. The prototype has been evaluated by a Dutch DSMH (i.e. sterility expert) of a Dutch sterilisation department (CSA services, The Meern, Utrecht, The Netherlands) who confirmed that proper cleaning of the system should be possible. Component inspection after the experiments indicated that the system can be used for at least 100 times without signs of wear or damage.

### Pilot study

**Fig. 7:**
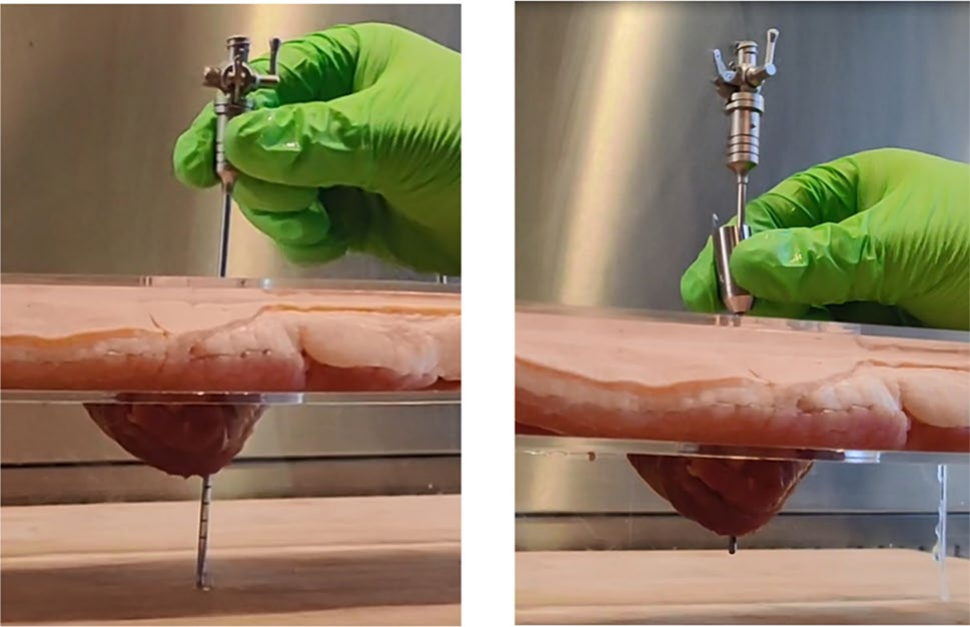
Typical end-situations after inserting the conventional VN (left) and VN + with decoupling mechanism (right)

**Fig. 8:**
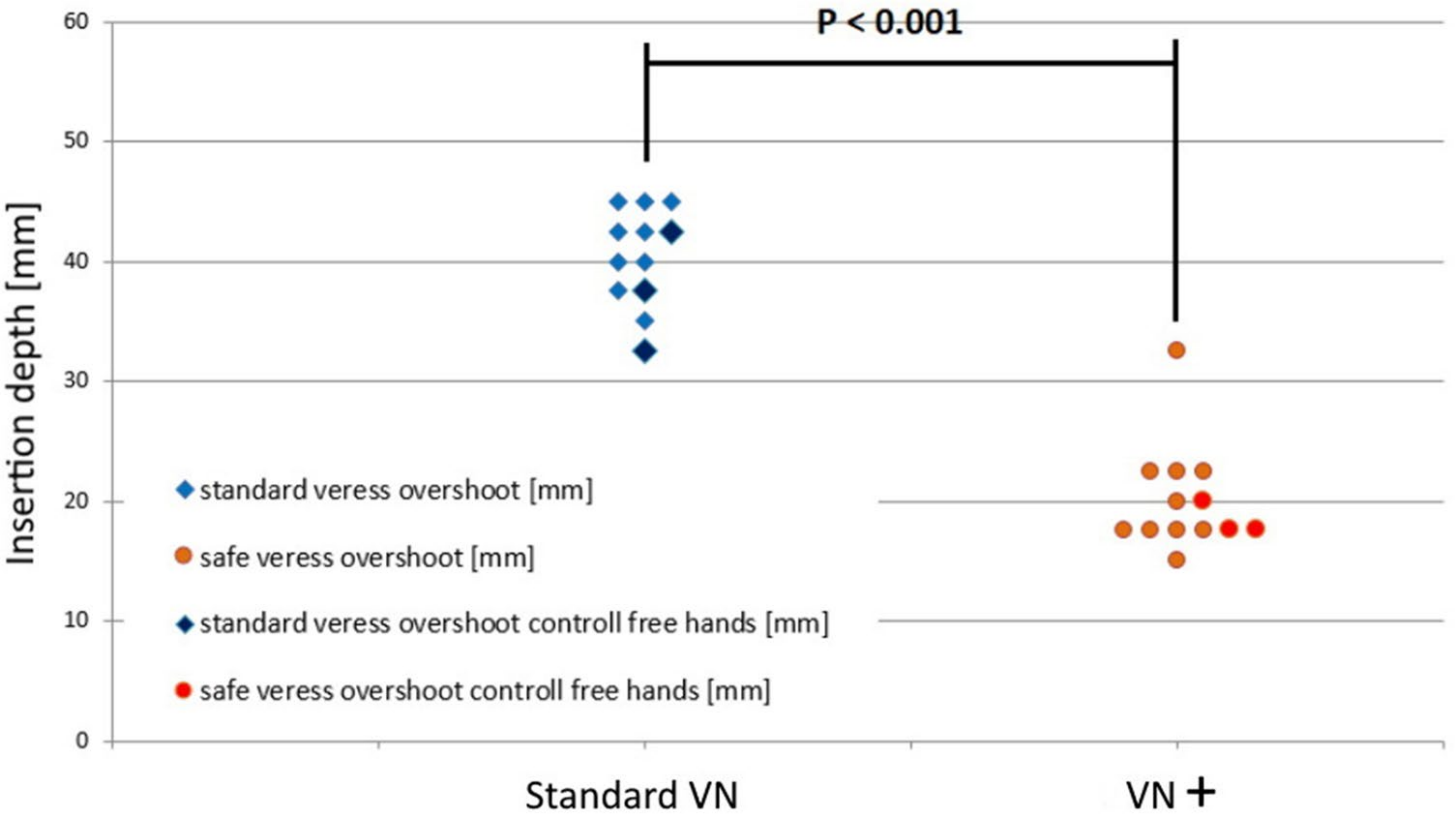
Pilot study data. Left, maximal insertion depth when using the standard VN. Right, maximal insertion depth when using the VN + with decoupling mechanism

Supplemental file 2a shows videos of the experiments conducted with the conventional VN and Supplemental file 2b shows videos with the VN+ with decoupling mechanism. The total experiment including the introduction, instructions and tasks took a maximum of 30 min per participant. Figure 7 shows typical end-situations after inserting the standard VN and VN+ with decoupling mechanism. Supplemental File 3 shows the total insertion depth data as presented in Fig. 8.

The Mann–Whitney test indicated a significant difference between the attempts with a standard, conventional working VN (41.4 mm [37.5–45 mm]) and VN+ with decoupling mechanism (20.8 mm [17.5–22.5 mm]) of *p* < 0.001. The participants did observe differences in force needed to penetrate the different layers and the number of force build ups during the insertion task. When using the conventional VN the wooden block under the setup was not touched by the needle tip. In the rest of the attempts with the standard VN the block was firmly hit and a sound was heard by the observers. When using the VN+ the wooden block was not reached by the needle tip. From the 26 attempts, only one attempt failed when the standard VN jammed and the inner cannula did not move back after penetrating the last layer due to tissue residue build-up between stylet and cannula.

## Discussion

A new decoupling safety mechanism was integrated successfully in a standard VN resulting in a VN+ that complies with all current cleaning requirements [28]. The mathematical models successfully related the forces acting on the flexor and hook to the flexor dimensions, indicating that the internal spring stiffness inside the housing of the VN and angle of the hook are important factors of influence. From a functional point of view the flexor should be as thin as possible. However, from a practical point of view the system should not be damaged during use or cleaning. Therefore it is advised to remain as close as possible to the maximum allowable flexor thickness during further optimisation.

The Pilot study data showed that the decoupling mechanism in the VN+ prevents the VN+ from accelerating inside the abdominal cavity as soon as the reaction force of the tissue suddenly drops to zero when the final tissue layer is penetrated. The additional decoupling mechanism is actuated by the original Veress mechanism when the blunt inner stylet pops out to protect the sharp needle tip of the cannula. Therefore, the VN+ expands on the original functions of the standard components in the VN without altering them in line with the Bare Minimum Design method for increased acceptance [26–28]. Although the diameter of the handle part of the VN+ was increased with 2 mm due to the additional grip component, none of the participants mentioned this during the evaluation of the system. Although an experienced CSSD sterility expert indicated that proper cleaning is possible, this was not yet tested in practice.

On average, the insertion depth of the VN+ was 20.8 mm. An optimisation step containing reduction of mass of the VN+ components and decrease of friction between components will most likely reduce insertion depth even further. During our pilot study the wooden shelf was hit with high force in nine of the ten attempts when using the conventional VN, but never with the VN+. Therefore, the actual insertion depth difference between the groups could be considerably higher if the overshoot was not limited by the test setup.

Changing direction of the insertion force during insertion should be prevented as this could activate the safety mechanism too early. Although this does not seem harmful, the surgeon needs to use the other hand to put the grip component back in position before continuation. The “free trial” session before the experiment with all the participants showed that there could be a small learning curve present. As soon as the participants started to trust the mechanism and were not afraid to apply force during insertion, the system did not result in early detachment anymore.

The results from the 6 additional trials conducted by the experienced surgeon on a loose abdominal wall sample did not differ from the earlier measurements. This indicates that the fixation of the abdominal wall did not influence the measurements. As the VN+ showed real potential during the pilot study, our team plans to fully characterise the system during a human cadaver study with representative tissue properties to ensure that all risks are accounted for.

Although the safety mechanism was integrated in a VN, other needle and trocars systems can benefit from this approach as well. In a war zone, lifesaving access systems, used to access major blood vessels to prevent or treat haemorrhagic shock or to establish access to the airway/trachea, are often used by less experienced medical personnel working under stressful situations. Further studies should indicate if integration of a similar safety mechanism in those lifesaving needle systems can reduce failure.

### Limitations

Since this was a preclinical evaluation several potential differences to the real clinical situation can be discussed. Some surgeons adopt different techniques to avoid overshooting when using the VN, e.g. placing the wrist of the hand on the patient’s skin and manipulating the VN only with the force of the fingers and wrist instead of the whole arm to reduce overshooting; slowing down between each layer of the abdominal wall to have higher control and to reduce overshooting. These strategies, in combination with conventional VN and VN+ , should be addressed in further usability studies. In addition, the current evaluations were performed on an ex vivo porcine composite abdominal wall model with standardized settings. Although the surgeon suggested that the porcine tissue properties felt realistic, the results will have to be transferred and validated in human cadaveric settings as well as live animal models before use of VN+ on patients. The use of the VN+ will also have to be assessed for different entry points to the abdomen such as “Palmers” point in the left subcostal region as well as midline and periumbilical regions and lower abdomen to account for different tissue resistance settings with regards to distance to bony- and other structures [7]. Many surgeons employ strategies like pulling up the skin with their hands or forceps to lift the abdominal wall away from abdominal organs to reduce risk of damage [29]. Additional comparison studies should indicate if this behaviour resembles the preclinical setting of the present study with a fixed size exit hole of several centimetres or that modification of the setup is needed to increase realism. Still, the current evaluations show enormous potential of VN+ for improving safe access to the abdomen for laparoscopic surgery by reducing overshooting and subsequent risk of organ damage. Therefore further development and investigation is warranted.

## Conclusion

The bare minimum design approach with focus on function expansion of existing components resulted in a new safety mechanism that was successfully integrated in an existing VN. The results from the pilot study indicate that this new VN+ reduces overshooting with a minimum of 50% in a standardised ex vivo setting on fresh porcine abdominal wall specimens. Optimisation could reduce the mass of the VN+ components and friction between components which could further reduce the insertion depth. Although the results are very promising, additional experiments on all known insertion locations and on other cadaver material are needed to investigate how the obtained results translate towards a more clinically real setting.

## Supplementary Information

Below is the link to the electronic supplementary material.

Supplementary file1 (XLSX 10 kb)

Supplementary file2 (MP4 35935 kb)

Supplementary file3 (MP4 40533 kb)

Supplementary file4 (XLSX 18 kb)
